# Replantation

**Published:** 1883-12

**Authors:** W. Finlay Thompson

**Affiliations:** London


### THE DENTAL REGISTER.

Vol. XXXVIII.] DECEMBER, 1883. [No. 12.

### COMMUNICATIONS.

### Replantation.

BY DR. W. FINLAY THOMPSON, LONDON.

Read before the International Medical Congress, London, Aug. 9th, 1881.

The subject of replanting teeth, although frequently brought before the dental and medical professions, does not seem to have been thoroughly investigated by either body; yet it is one in which of late so many instances of treatment, successful or otherwise, have been recorded, that its discussion is not only permissible, but even necessary; and as the disquisitions of this Congress will undoubtedly bring it into more prominent favor, or tend to cast it into oblivion, I submit the following questions, hoping that they may bring out a full expression concerning it:—

I. Shall the system of replanting teeth, under any circumstances, be a recognized operation in dentistry? And, if so, under what pathological condition should this means be employed?

II. What advantages, in contradistinction to the disadvantages, may be claimed for replanting? Also, what percentage of cases are successful?

Before entering upon the practical bearings of the subject, it would be well perhaps to briefly consider the structure of a membrane upon which replantation is wholly dependent for its success.

The peridental membrane is an organized tissue which must be considered under two aspects—its relation to the vascular alveoli, and to the non-vascular cementum of the teeth. Its office, however, is similar under either aspect, as it acts as a periosteum or nutrient membrane to both, though modified in its action, and even in its texture, to a degree commensurate with the difference existing between the compact cementum and the soft cancellated alveolar tissue. Owing to the intimate relation of the peridental membrane to these two sections, it may—in the absence of pulp irritation—be considered the important factor in lesions connected with the teeth. It is chiefly composed of white fibrous connective tissue, and has a rich nerve and blood supply, while nucleated masses of protoplasm are to be found in every part. The wavy character of the fibres is more pronounced when seen in longitudinal than in transverse section. Their general course is oblique from the bony alveolus to the cementum, without break or interruption. According to Waldeyer, the nerve supply is abundant. The larger blood-vessels ramify in a general manner; those from the gum and alveolar surface also anastomose in its substance. The smaller masses of protoplasm are chiefly connective tissue corpuscles, while those near the bone and cementum form the osteoblasts. It is to the vitality and functions of these histological elements in particular that we must have the greatest regard in considering or treating any pathological condition of the dental perisoteum.

Reverting to the first question, "Shall the system of replanting teeth, under any circumstances, be a recognized operation in dentistry?" The recorded instances of success in replanting have perhaps been looked upon as evidences of a certain power in Nature not practically available, and consequently classed with other curious anomalies in the transposition of tissues. Want of success, though operating under incompatible conditions, has discouraged some from following to its conclusion a method which certainly has, in some instances, been practiced advantageously. These remarks must not be misunderstood; for I contend that the system of replanting should not be introduced in practice, save in severe and otherwise hopeless cases, and it is to these alone that reference is now made.

In regard to the physiological action in the reunion of the tooth with the alveolus, we can only reason from analogy. The evidences, however, of the existence of a perfect reticulum of connective tissue with fibres and protoplasmic cells are abundantly proved.

In fact, the osteoblasts accumulate in such numbers on the surface of the cementum, that, in certain inflammatory disorders, they act antagonistically, removing part of the cement, producing characteristic striæ and depressions. The office of protoplasm in the reparation of dissevered tissues is so well known that I need only call attention to the existence of this germinal matter in the peri-cementum to at once establish a *prima facie* evidence of the probability of the reunion of a tooth with its socket, through the intervention of the alvealo-dental membrane. As we see by an examination of the peri-cementum, there are, in the healthy condition of the tooth, immense numbers of masses of living matter in connection with the various parts entering into its composition. In health, these germs go through their proper changes and perform their proper functions. In diseased conditions, or in any departure from the healthy state, these protoplasmic bodies are also the principal agents, and, in such departures from the healthy state, they are excited to action by excessive supply of pabulum, which causes each individual mass of living matter to live faster than it should—*i.e.*, it takes up more pabulum and converts this into more living matter like itself than it does in the normal condition; it becomes enlarged, it divides and sub-divides, and multiplies to such a degree that, in the place of the healthy tissue, we have a pus formation. The protoplasmic bodies in the blood—or white blood corpuscles—also actively assist in the inflammatory process. In recorded cases, it is stated that teeth have been successfully replanted without peri-cementum upon them. The means of union in such cases I shall not now discuss, but, at the end of my paper, this subject will be indirectly included in a question I shall then put to the meeting; yet it may not be without interest if I cite a case which lately came under my care, where the return of the tooth to its socket was, from various causes, delayed for twenty-five hours. In this interval, instead of being kept in a solution of salt and water, it was encased in bibulous paper moistened with carbolized water. The tooth, after being returned to its socket, appeared for a time to progress favorably; but was, notwithstanding, ultimately lost. It is a question whether this destruction of vital power was not in consequence of too long an interval between extraction and replanting, or whether the carbolized water destroyed the protoplasmic germs which were to, in part, restore vascular circulation between the alveolus and the peri-cementum. I infer that, in the necrosed condition of the periosteum, which was not tolerated by the plastic effusion from the alveolus, sloughing resulted, rather than any fibrous connection with the cementum

There is, even in successful cases, great difference in the stability of replanted teeth. In some, considerable mobility exists, indicating a lax fibrous attachment; this sometimes occurs in undrained teeth, caused, no doubt, by the retention of septic matter, or effusion of inflammatory products into the connective tissue. In others the fixed condition of the tooth suggests anchylosis. These cases are usually found where drainage has been provided for, thus permitting egress of the morbid accumulations from suppuration, as well as the sequelæ of organizing inflammation.

Whether the inflammation be acute or chronic, the practice of replantation demands parallel procedure—viz., the removal of all the disintegrated periosteum, the destruction of the sac at the apex of the root, and the careful polishing of the cementum where the osteoblasts, from inflammatory influence, have reversed their function and begun to reabsorb the osseous structure of which they were originally formative. This process may vary in its intensity from merely causing microscopic pits in and prominences on the cementum, to the dissolution of that tissue, and ultimate wasting of the root of the tooth. It was this condition which suggested to me a possible advantage of capping. Of the subject of capping I shall shortly treat.

II. What advantages, in contradistinction to the disadvantages, may be claimed for replanting? Also what percentage of cases are successful?

Those who have treated chronic alveolar abscess must, at times, have experinced the uncertainty of their efforts. Day after day, perhaps for weeks, and even months, have patients been subjected to the inconvenience of almost daily attendance upon their dentists, with but doubtful benefits being conferred, resulting, perhaps, in only temporary retention of the tooth. Shall we not consider this a case open for the adoption of any means promising relief? Then, again, there may be conditions under which the time required cannot be devoted to the case, as often happens when parties come from a distance. If treatment calculated to secure a valuable organ can be performed in a single sitting, shall we not deem it a justifiable proceedure?

The operation is one which, from its very nature, must be brought to a quick conclusion. Although, between the intervals of extracting and restoring the tooth again to its socket, several hours may elapse, no unnecessary time should be wasted in its return.

It may be alleged that the severance of the contiguous tissues, and the disrupture of the nutrient vessels, is of such a character as to preclude solid reunion of the cementum, of the peri-cementum, and the gum and alveolus. That the reunion may partake of the nature of a "seamed scar," and therefore be so far incomplete, I shall not attempt to refute; but that it is sufficient to continue the low vitality required to ensure the retention of the tooth is now fairly well established by the lapse of years in cases of replanted teeth, which are apparently sound and useful to this day. As an instance:—

A patient of the late Mr. Edwin Sercombe, who came under my care for general treatment of the mouth, gave me the history of a second inferior bicuspid replanted some ten years before by that gentleman. The tooth was not discolored, and it had every appearance of being alive. A slight retrocession of the gum and alveolus had taken place, but not to such an extent as to cause any inconvenience. Numerous other equally successful cases have been recorded.

Perfect restoration of the tooth can also be made where ugly forms of decay extend under the gum to almost inaccessible positions, with a perfection limited only by the skill of the operator, as his ability is not then dependent on the endurance of the patient, whose irritability ceases to be an element in the difficulties of the operation. This advantage, however, should in no way bias a practitioner in the treatment of any case.

The extraction, performed under the influence of nitrous oxide, is of course, painless; and when the manipulation of the tooth is complete, gas may again be called into requisition in this the only part of the operation from which any further acute pain may arise. Once the tooth is established in position and carefully adjusted in respect to its occluding surfaces, rarely more than tenderness and uneasiness of a few days' duration are experienced.

Having viewed one side of the question, I shall proceed to consider the disadvantages that may be claimed in opposition to it.

We have to combat the antagonism which naturally arises from the mere mention of an operation, the peculiar nature of which is repugnant to the feelings of the patient. From the limited general information, or, perhaps, lack of personal experience in connection with the subject, its results are undoubtedly, at the present time, sceptically received by the profession.

The nutrient structural attachment being ruptured, time will be required to establish reunion of the parts. For several days the tooth must be considered an object of constant care by the patient, who will occasionally be annoyed by its occlusion with the opposing teeth. Although uneasiness may be experienced as an accompaniment to the operation, it is doubtful whether the actual pain is in excess of that caused by local applications.

The next restraining influence in the practice of replanting, is the uncertainty and known danger of extraction; this is hemmed in with circumstances quite beyond control. The shape of the alveolus and of the tooth would, in many instances, enable a favorable prognosis of extraction, yet in some cases it is impossible to accurately predict the result, for the divergence or distortion of the roots may be so great as to cause fracture of the alveolus or of the root itself, while this same distortion may preclude the return of the organ to its socket.

In considering "What percentage of cases are successful?" I shall first briefly describe the system of treatment which I have adopted. In cases of chronic abscess and where the apex of the root has lost its periosteum, the denuded portion of the root has been excised, and restored with a gold cap, the object being, if possible, to prevent absorption. To remove inflammatory products, instead of drilling through the alveolus, or cutting a groove along the roots, a tube has been fixed in the pulp canal, extending from the apex of the root to the grinding surface. From my experience I do not now ascribe much of the success that I have had to the cap; but the tubing, inasmuch as it permits of drainage, I deem important. How far the cap has met the object for which it was intended, I am unable at present to give any reliable information, simply from the fact that, in every case where the tooth has been retained for a period of over fifteen days, I *have had no opportunity of again seeing it.* That this foreign substance has been tolerated by the living tissues is a well-authenticated fact, the physiological effects of which must for a time rest in abeyance.

I have spoken of the difficulty, in some instances, of returning teeth to their sockets. A case illustrative of this came under my care some two years since, the tooth being a superior first molar, and the palatal root standing at an angle of about 35°. The tooth having for a long time been affected by chronic abscess, and the lady being non-resident in London, with only a limited time which could be devoted to treatment, I decided to extract, fill, and replant it. The tooth was removed at twelve o'clock and returned at seven P.M. same day. Three-fourths of the crown had been destroyed by decay, and the post-proximate aspect was broken down far below the margin of the gum. This is undoubtedly a case that I should have failed in again restoring to the mouth had it not been for the kindness of Mr. Woodhouse Braine, who rendered me valuable assistance in keeping the patient under the influence of gas while it was both taken out and returned to its socket. I make particular mention of the gas in this case because I was not able to re-establish the tooth in its position until I had used almost the same force in its return that I did in extracting it. The lady called some time since, and, having occasion to take nitrous oxide again, it gave Mr. Braine an opportunity to examine the condition of the tooth. The lady stated that it had done the best of service; and, after the first few days, had occasioned her no discomfort. In this case the tooth seems fixed—a condition that I am not prepared to say is an advantage.

During a visit of Dr. Wm. N. Morrison, to London, replantation was a subject of considerable discussion between him and myself. I was endeavoring to investigate the practical results (if any) that might be obtained by this method; and my conclusions are drawn from results extending over a period of three years. For the first two years I made it a special point of interest to obtain as many cases as possible. During the last year, however, I have only replanted four teeth. My observations have been made principally upon people in the lower walks of life. Many cases I have completely lost sight of, so that my failures may not all be recorded.

| | |
|---|---|
| Number of teeth replanted (estimated) | 80 |
| Lost, as far as known | 8 |

The losses were as follows:—

| | |
|---|---|
| From non-union | 5 |
| Imperfect union; three months' retention | 1 |
| Abscess recurring | 2 |
| Lost cases, as far as known | 8 |

Of the eight lost, five were not tubed. Of the remaining three tubed, one abscessed again, and the other two were lost from non-union.

Of the number of teeth replanted, I am not able to state the exact proportion tubed, but I estimate it as something over 50 per cent.

During the last twelve months, the number of teeth replanted by me has been:—

| | |
|---|---|
| In private practice | 1 |
| Hospital patients | 3 |
| | 4 |

Before closing, I desire to call attention to the subject of transplantion of tissues; and, through the kindness of Mr. C. Macnamara, Surgeon to the Westminster Hospital, I am permitted to refer to a case which has been of considerable interest to me. Mr. Macnamara seems to have been influenced in the treatment adopted by what he had seen in connection with teeth replanted. It is (so far as I know) the second instance only in which transplanting of bone has been effected of the same nature and of so extensive a character, and, thinking that it might possess features of interest to this Congress, I have, with the consent of Mr. Macnamara, arranged for as many to see the case as would like to do so. The clinical notes handed to me are as follows:—

"S. W., aged 6, was admitted into the Westminster Hospital, August 10th, 1880, suffering from necrosis of the upper extremity of the right tibia. The inflammation extended to, and destroyed, the shaft of the bone. Mr. Macnamara consequently, on June 2nd, 1881, removed the dead bone, that is, the entire shaft of the tibia, the upper and lower epiphyses being left in situ, and as much of the periosteum as could be saved. The wound healed, but no new bone formed, so that the child's leg was useless.

"On May 21st, 1881, Mr. Macnamara determined, if possible, to plant small fragments of bone in the situation of the lost tibia, in the hope that they would produce a new bone. Having a case of amputation of the foot for deformity, the bones of the tarsus were, immediately after its separation from the body, divided into small pieces, and an incision having been made along the front of S. W.'s leg, from the upper to the lower epiphysis, fragments of bone were placed along the situation occupied by the former tibia; the edges of the wound being then brought together. The operation was done under the carbolic spray and antiseptic dressings.

"Two days after the operation, on removing the dressings, there was found to be some tension about the wound, and the sutures were consequently divided and taken away. On the third day, several of the fragments of bone planted in this child's leg were seen to be vascular and attached to surrounding living structures; they have since grown, and are now covered in by granulations. So far as can be ascertained at present, there seems good hope of sufficient osseous tissue remaining and growing in the leg to produce a new tibia."

In sub-periosteal operations the physiological changes that take place in the membrane to elaborate new bone seem almost incomprehensible. This reproductive power was strikingly evinced in another case, the particulars of which have been kindly furnished to me by Mr. Macnamara.

"A. L. was admitted into Westminter Hospital under my care on the 26th of March, 1879, with acute necrosis of the upper extremity of the right tibia, and consequent septicæmia, from which the lad remained in a most critical position for nearly a month. He then got so far well as to enable me either to ampu tate the limb or to take away the shaft of the tibia without removing the periosteum. I determined on the latter operation, and the section of the shaft of the tibia removed is exhibited in the Museum of the International Medical Congress, No. 19. The boy recovered well from the operation; and, from the perisoteum I left in his leg, not only has a new tibia grown, but the bone is larger than we know what to do with. The patient has got a good knee and ankle-joint, and can walk about perfectly well."

These two cases possess many points of interest which bear upon the subject of replantation of teeth. One question, at least, I should wished discussed here, is, what points of analogy or of difference are there between the nature of the repair and of bone-formation in such instances as these cited by Mr. Macnamara, and the repair and re-attachment which takes place between the bone of alveolus and the cementum of a replanted tooth?

The vivacity of the periosteum, and the reparative action it is shown to be the seat of, together with the pathological and even destructive functions, it is capable of performing, renders a more perfect knowledge of the structure of that tissue, in its several positions and relations, a matter of the greatest importance to prevent error even in such operations as the replantation of teeth.

